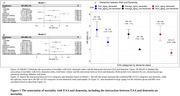# Accelerated aging elevates the risk for mortality in persons with dementia: the Age, Gene/Environment Susceptibility‐ Reykjavik Study cohort

**DOI:** 10.1002/alz.089435

**Published:** 2025-01-09

**Authors:** Nigus G. Asefa, Zhiguang Li, Yi‐Han Hu, Jorge M. Romero, Osorio Meirelles, Vilmundur Gudnason, Lenore J. Launer

**Affiliations:** ^1^ Laboratory of Epidemiology and Population Sciences, National Institute on Aging, BALTIMORE, MD USA; ^2^ National Institute on Aging, Baltimore, MD USA; ^3^ Laboratory of Epidemiology and Population Sciences, National Institute on Aging, Baltimore, MD USA; ^4^ national institute on aging, BALTIMORE, MD USA; ^5^ Icelandic Heart Association, Kopavogur Iceland; ^6^ Faculty of Medicine, University of Iceland, Reykjavik Iceland

## Abstract

**Background:**

Epigenetic age acceleration (EAA) is a valuable tool for predicting all‐cause mortality and assessing disease risk. Differences in EAA reflects biologic aging (BA) and suggests underlying differences in morbidities. We examined whether EAA moderated the association of dementia to mortality risk.

**Method:**

Analyses are based on data from the Age, Gene/Environment Susceptibility‐ Reykjavik Study (AGES‐RS) study (n = 2602, 57.5% females, mean age = 75.8 years, 30% demented). Data were collected from 2002 to 2006, with mortality data available until 2015. EAA was computed with the DunedinPACE algorithm and grouped as follows: EAA scores > 1 SD of the mean were labeled as ‘fast’, EAA < 1 SD as ‘slow’, and EAA scores within ±1 SD as ‘average’. Dementia was ascertained in a study exam or through follow‐up of health records. Cox proportional hazard analysis was used to evaluate the hazard ratio (HR, 95%CI) of mortality and the interaction between EAA and dementia. Models were adjusted for sex, chronological age, education, smoking, diabetes and stroke.

**Result:**

Fast agers had higher mortality HRs than average agers (Figure 1A). Fast agers with dementia had a significantly increased risk of death (interaction p‐value = 4.08×10^−2^) compared to non‐demented average agers (Figure 1C); this interaction was reduced after adjusting for total brain volume (Figure 1B). Conversely, slow aging was associated with reduced mortality risk (HR = 0.71, 95% CI: 0.52‐0.98, p‐value = 3.62×10^−2^); however slow agers with dementia had risk estimates similar to non‐demented average agers (interaction p‐value = 1.14×10^−1^) but lower than demented fast agers. Controlling for total brain volume reduced the differences between slow and average agers with and without dementia.

**Conclusion:**

Individuals aging at a faster rate than their chronological age were at a higher risk for death. The rate of BA had an effect on the association between dementia and mortality. The association was partially explained by brain atrophy, suggesting other factors related to dementia contributing to mortality risk in people with dementia.